# Imipenem/cilastatin/relebactam treatment of hospital-acquired/ventilator-associated bacterial pneumonia caused by imipenem-nonsusceptible pathogens: Subgroup analysis and molecular characterization of isolates from the RESTORE-IMI 2 clinical study

**DOI:** 10.1371/journal.pone.0357288

**Published:** 2026-09-15

**Authors:** Katherine Young, C. Andrew DeRyke, David W. Hilbert, Maria C. Losada, Jiejun Du, Amanda Paschke, Luke F. Chen

**Affiliations:** Merck & Co., Inc., Rahway, New Jersey, United States of America; Nitte University, INDIA

## Abstract

**Background:**

Additional treatment options are needed for patients with hospital-acquired and ventilator-associated bacterial pneumonia (HABP/VABP) caused by carbapenem-resistant pathogens. This subgroup analysis examined clinical and microbiologic outcomes in participants from RESTORE-IMI 2 treated with imipenem/cilastatin/relebactam (IMI/REL), stratified by baseline lower respiratory tract (LRT) isolate susceptibility to imipenem. Emergence of nonsusceptibility to IMI/REL during treatment was also evaluated.

**Methods:**

Baseline LRT specimens were obtained ≤48 hours before screening, identified at a central laboratory, and susceptibility determined per the Clinical and Laboratory Standards Institute breakpoints. Outcomes for participants who received IMI/REL, with ≥1 imipenem-nonsusceptible, imipenem/REL-susceptible baseline LRT pathogen were compared with participants with imipenem-susceptible pathogens. Emergence of nonsusceptibility to imipenem/REL was also assessed, and isolates were characterized molecularly.

**Results:**

In total, 215 participants in the microbiologic-modified intent-to-treat population received IMI/REL, of whom 112 were infected with imipenem-susceptible pathogens and 18 with imipenem-nonsusceptible, imipenem/REL-susceptible pathogens and were included in this analysis. Of these 18 participants, 88.9% were in the intensive care unit and 83.3% had one gram-negative baseline pathogen. Day 28 all-cause mortality was 22.2% and 18.8% (adjusted difference: 7.3 [−8.8 to 31.1]) for participants who received IMI/REL for the treatment of HABP/VABP caused by imipenem-nonsusceptible versus imipenem-susceptible pathogens, respectively. Of a total of 192 isolates, 59 gram-negative isolates (baseline isolates that were nonsusceptible to imipenem and subsequent isolates of the same species from the same participants) were characterized using multiplex polymerase chain reaction and sequencing techniques. Emergence of nonsusceptibility to imipenem/REL occurred in isolates from three of the 112 participants with indicated imipenem-susceptible baseline pathogens (two isolates of *Pseudomonas aeruginosa* and one isolate of *Klebsiella pneumoniae*).

**Conclusions:**

Day 28 all-cause mortality was similar among participants treated with IMI/REL, regardless of baseline pathogen susceptibility to imipenem. Emergence of nonsusceptibility to imipenem/REL on treatment was low.

**Clinical trials registration:**

ClinicalTrials.gov (NCT02493764).

## Introduction

Infections caused by carbapenem-resistant gram-negative bacteria are urgent global public health threats owing to a lack of antibacterial agents that are effective against these rapidly emerging pathogens [1–3]. Carbapenem-resistant isolates can be co-resistant to other first-line antibacterial agents, such as piperacillin/tazobactam (PIP/TAZ) and cefepime [4–6]. Infections caused by carbapenem-resistant gram-negative pathogens are associated with worse treatment outcomes than infections caused by carbapenem-susceptible pathogens [7]. Resistance to carbapenems almost always involves β-lactamases, which can hydrolyze the antibacterial agent, thereby rendering it inactive [8,9].

Several antibacterial agents have been developed to treat infections caused by carbapenem-resistant gram-negative pathogens [10–12]. Imipenem/cilastatin/relebactam (IMI/REL; RECARBRIO) is a carbapenem/β-lactamase inhibitor combination approved in the United States and the European Union for the treatment of hospital-acquired and ventilator-associated bacterial pneumonia and infections caused by aerobic gram-negative pathogens in adult patients with limited treatment options, such as those with complicated urinary tract infections (cUTIs) or complicated intra-abdominal infections (cIAIs). It is also approved in the European Union for treating bacteremia associated with hospital-acquired bacterial pneumonia (HABP)/ventilator-associated bacterial pneumonia (VABP) [13,14]. The results of the Phase 3 RESTORE-IMI 2 study demonstrated that IMI/REL was non-inferior to PIP/TAZ for treating HABP/VABP in terms of both day 28 all-cause mortality (ACM) and favorable clinical response at the early follow-up (EFU) visit [15]. We undertook this subgroup analysis to understand whether outcomes differed among patients treated with IMI/REL whose baseline pathogen was imipenem-nonsusceptible compared with patients whose baseline pathogen was imipenem-susceptible. We also evaluated the emergence of nonsusceptibility to IMI/REL through day 28, as well as the molecular characteristics of those isolates.

## Materials and methods

### Overall trial design

RESTORE-IMI 2 (NCT02493764; protocol 014) was a Phase 3, randomized, double-blind, multicenter study of adult participants from 113 hospitals in 27 countries with HABP/VABP treated with either 500/500/250 mg IMI/REL or 4 g/500 mg PIP/TAZ intravenously every 6 hours for 7–14 days. Trial results and details of the trial methodology have been described previously [15]. In brief, specimens were collected from November 2015 through January 2016, and molecular characterization of samples occurred from June 2019 through August 2019. Baseline lower respiratory tract (LRT) specimens were obtained ≤48 hours before screening and bacterial isolates were identified at a central laboratory. Additional samples may have been taken during treatment (OTX), at end of treatment (EOT), and at EFU (7–14 days after EOT; up to 31 days post-randomization). The RESTORE-IMI 2 study was conducted in accordance with the principles of Good Clinical Practice and was approved by the appropriate institutional review boards and regulatory agencies. Written informed consent was obtained from all participants prior to the study start and included the use of the specimens used in the present analyses.

### Susceptibility testing and molecular characterization

Broth microdilution of imipenem/REL was performed following Clinical and Laboratory Standards Institute (CLSI) methodology [16], using a fixed 4 µg/mL concentration of relebactam. Nonsusceptibility to imipenem/REL was determined using the following CLSI breakpoints for minimum inhibitory concentration (MIC): > 1 μg/mL for non-*Morganellaceae* Enterobacterales (NME) and >2 μg/mL for *Pseudomonas aeruginosa* [16].

Of LRT gram-negative isolates collected at any visit throughout the trial, those that were either imipenem-nonsusceptible or imipenem/REL-nonsusceptible underwent molecular characterization (i.e., evaluation for the presence of resistance determinants against β-lactam agents using multiplex polymerase chain reaction [PCR] and sequencing; S1 and S2 Fig) [17,18]. Baseline isolates were also characterized for participants who yielded imipenem-susceptible or imipenem/REL-susceptible isolates at baseline and subsequently yielded imipenem-nonsusceptible or imipenem/REL-nonsusceptible isolates during the study, respectively. The methods have been described previously [17,18] and were used to characterize the isolates as encoding one or more of the following: extended-spectrum β-lactamases (ESBLs), *Klebsiella pneumoniae* carbapenemases (KPCs), *Pseudomonas*-derived cephalosporinases (PDCs), oxacillinase-48 (OXA-48)-like carbapenemases, Ambler class C cephalosporinases (AmpC), and/or metallo-β-lactamases (MBLs).

Key pathogens included the following, based on their epidemiological association with HABP/VABP and susceptibility to imipenem/REL: *Acinetobacter calcoaceticus–baumannii* complex, *Citrobacter freundii*, *Citrobacter koseri*, *Enterobacter cloacae*, *Escherichia coli*, *Haemophilus influenzae*, *Klebsiella aerogenes*, *Klebsiella oxytoca*, *K. pneumoniae*, *P. aeruginosa,* and *Serratia marcescens* [13,14]. The emergence of nonsusceptibility to IMI/REL was defined as any key pathogen susceptible to imipenem/REL at baseline, but nonsusceptible to imipenem/REL at any time post-baseline through day 28. This also included post-baseline isolates with a 1 dilution change in MIC as compared with the baseline isolate (intermediate susceptibility), which is often considered to be within the limits of experimental variability; however, here we conservatively interpreted this increase as emergence of nonsusceptibility (intermediate susceptibility or resistant). Emergence of imipenem- or imipenem/REL-nonsusceptible isolates during treatment was analyzed further by β-lactamase analysis, porin profiling, and pulsed-field gel electrophoresis (PFGE).

### Imipenem-susceptible and imipenem-nonsusceptible subgroup analyses

This analysis of the protocol-defined microbiologic-modified intent-to-treat (mMITT) population included all randomized participants with ≥1 dose of IMI/REL and ≥1 baseline bacterial pathogen identified as the cause of HABP/VABP against which IMI/REL is known to have antibacterial activity, excluding those participants with only gram-positive cocci present on baseline Gram stain. Two subgroups of the mMITT population were retrospectively defined by CLSI breakpoints [16]. The imipenem-nonsusceptible group comprised all participants with ≥1 baseline LRT pathogen that was nonsusceptible to imipenem and susceptible to IMI/REL. The imipenem-susceptible group comprised all participants with baseline pathogens that were susceptible to both imipenem and imipenem/REL. For any pathogens without CLSI susceptibility interpretative criteria [16] for imipenem/REL, imipenem breakpoints were applied. All participants with baseline LRT pathogens that were nonsusceptible to both imipenem and imipenem/REL were excluded from this analysis.

In this subgroup analysis, the following endpoints were evaluated retrospectively: day 28 ACM (primary endpoint of the trial), clinical response at EFU (7–14 days after EOT; key secondary endpoint), and microbiologic response at EOT (secondary endpoint). A favorable clinical response at EFU was indicated by an assessment of ‘cure’, ‘sustained cure’, or ‘improved’. A favorable by-pathogen microbiologic response at EOT was indicated by an assessment of ‘eradication’ of the baseline pathogen.

## Results

Among participants treated with IMI/REL in RESTORE-IMI 2 based on fulfillment of clinical and radiographic criteria for HABP/VABP and the detection of gram-negative bacteria upon evaluation of a LRT specimen, 215 were included in the mMITT population. Of these, 112 were included in the subgroup with imipenem-susceptible isolates and 18 were included in the subgroup with imipenem-nonsusceptible isolates; 85 of the 215 participants were excluded from this analysis because they were infected with organisms that were nonsusceptible to both imipenem and imipenem/REL (e.g., *A. calcoaceticus–baumannii* complex or *Stenotrophomonas maltophilia*). Baseline demographics and clinical characteristics were generally comparable between the imipenem-susceptible and imipenem-nonsusceptible groups (Table 1). Both *K. pneumoniae* (44.4%) and *P. aeruginosa* (33.3%) were more prevalent in the imipenem-nonsusceptible group compared with the imipenem-susceptible group (27.7% and 16.1%, respectively).

**Table 1:** Baseline participant demographics, clinical characteristics, and pathogens by imipenem susceptibility subgroup (mMITT population).

Characteristic	Imipenem-nonsusceptible group (N = 18)	Imipenem-susceptible group (N = 112)
Age, median (range), years	60.0 (41‒89)	63.0 (18‒95)
≥65, n (%)	7 (38.9)	51 (45.5)
Male sex, n (%)	12 (66.7)	81 (72.3)
White race, n (%)	13 (72.2)	89 (79.5)
APACHE II score ≥15 at baseline, n (%)	8 (44.4)	54 (48.2)
Pneumonia type and primary diagnosis, n (%)		
Non-ventilated HABP	11 (61.1)	54 (48.2)
Ventilated HABP	2 (11.1)	11 (9.8)
VABP	5 (27.8)	47 (42.0)
In the ICU, n (%)^a^	16 (88.9)	77 (68.8)
Mean CrCl, mL/min (SD)	89.5 (38.7)	104.2 (65.6)
Renal function at baseline, n (%)		
Normal (CrCl ≥ 90 mL/min)	10 (55.6)	59 (52.7)
Mild RI (CrCl ≥ 60 to <90 mL/min)	3 (16.7)	21 (18.8)
Moderate RI (CrCl ≥ 30 to <60 mL/min)	5 (27.8)	27 (24.1)
Severe RI (CrCl ≥ 15 to <30 mL/min)	0	5 (4.5)
Baseline pathogens, n (%)		
Subjects with one baseline pathogen	15 (83.3)	102 (91.1)
Subjects with ≥2 baseline pathogens	3 (16.7)	10 (8.9)
Pathogens identified		
*Acinetobacter calcoaceticus–baumannii* complex	1 (5.6)	2 (1.8)
*Klebsiella aerogenes*	1 (5.6)	4 (3.6)
*Klebsiella pneumoniae*	8 (44.4)	31 (27.7)
*Pseudomonas aeruginosa*	6 (33.3)	18 (16.1)
*Haemophilus influenzae*	3 (16.7)	7 (6.3)

^a^Participants in the ICU at randomization.

APACHE, Acute Physiologic Assessment and Chronic Health Evaluation; CrCl, creatinine clearance; HABP, hospital-acquired bacterial pneumonia; ICU, intensive care unit; mMITT, microbiologic-modified intent-to-treat; n, number of patients with at least one of the baseline pathogen; N, number of mMITT patients; RI, renal impairment; SD, standard deviation; VABP, ventilator-associated bacterial pneumonia.

**Fig 1:**
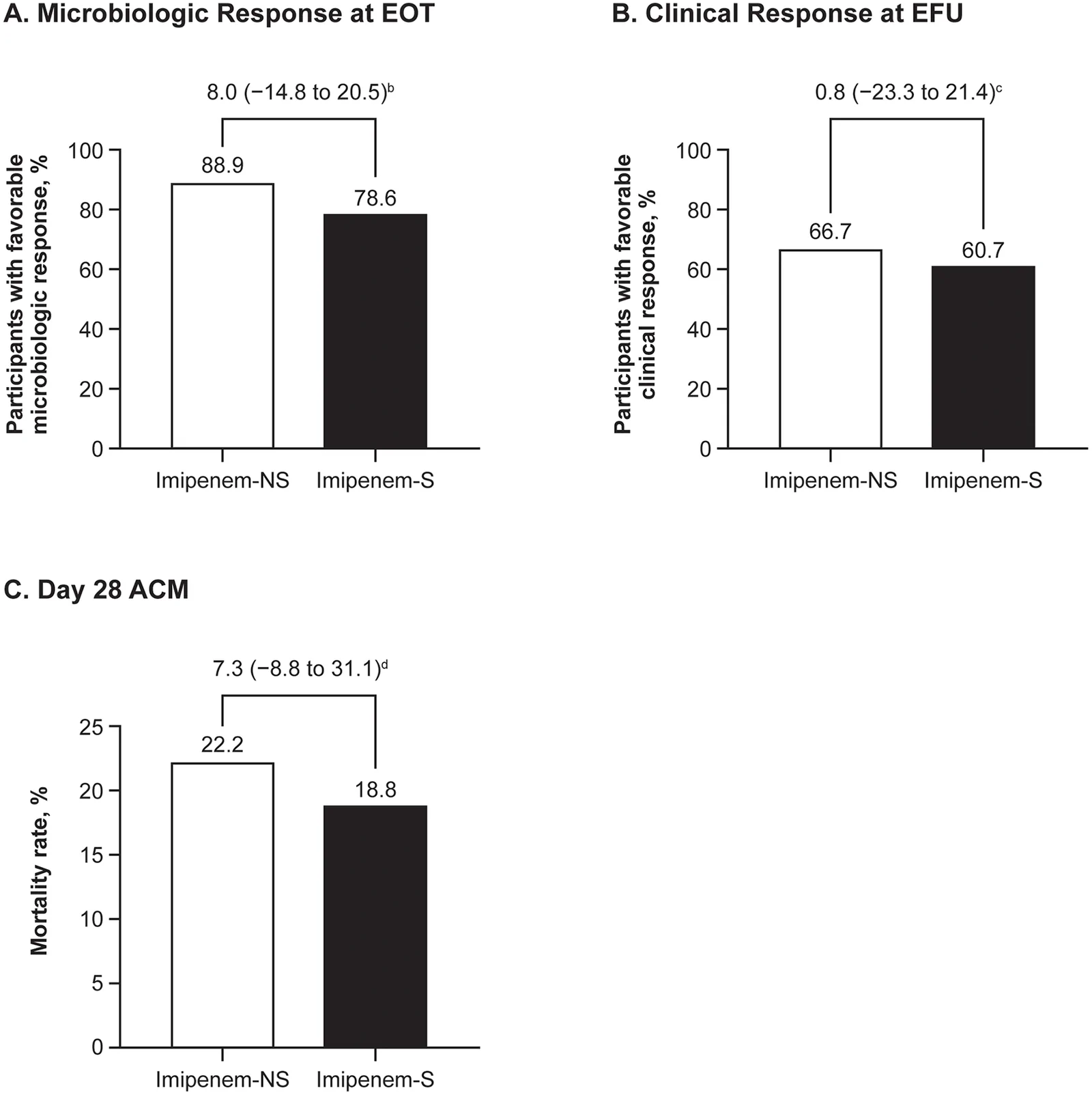
Treatment outcomes of participants treated with IMI/REL stratified by imipenem susceptibility of baseline LRT pathogens (mMITT population)^a^: favorable microbiologic response (A), favorable clinical response (B), and day 28 ACM (C). ^a^Adjusted differences and the corresponding CIs are based on Miettinen & Nurminen method stratified by randomization stratum. ^b^95% CI. ^c^The 95% CI and the p-value are for the non-inferiority hypothesis test in which the non-inferiority margin was −12.5% and alpha level (1-sided) was 0.025. ^d^The 95% CI and the p-value are for the non-inferiority hypothesis test in which the non-inferiority margin was 10% and alpha level (1-sided) was 0.025. ACM, all-cause mortality; CI, confidence interval; EFU, early follow-up; EOT, end of treatment; IMI/REL, imipenem/cilastatin/relebactam; LRT, lower respiratory tract; mMITT, microbiologic-modified intent-to-treat; NS, nonsusceptible; S, susceptible.

Of the 18 participants who had isolates that were imipenem-nonsusceptible at baseline, 12 had a favorable clinical response at EFU, 16 had a favorable microbiologic response at EOT, and 14/18 were alive at day 28 (Table 2). Efficacy outcomes, including day 28 ACM, were similar between both groups of participants treated with IMI/REL, regardless of the susceptibility of baseline pathogens to imipenem (Fig 1). Day 28 ACM was 22.2% compared with 18.8% (adjusted difference: 7.3 [−8.8 to 31.1]) in the imipenem-nonsusceptible group and imipenem-susceptible group, respectively. Clinical responses at EFU in the imipenem-nonsusceptible subgroup were also similar to the imipenem-susceptible subgroup (66.7% vs 60.7, respectively), as were microbiologic responses at EOT (88.9% vs 78.6%, respectively).

**Table 2:** MICs, molecular characterization, and corresponding clinical outcomes for imipenem-nonsusceptible baseline LRT pathogens from the IMI/REL treatment subgroup.

LRT pathogen	Imipenem MIC (µg/mL)	IMI/REL MIC (µg/mL)	Beta-lactamases	Microbiologic response at EOT	Clinical response at EFU	Day 28 ACM
*Klebsiella pneumoniae*	2	1	*SHV-1; TEM-1; CTX-M-15; OXA-48*	Favorable	Favorable	Alive
*Klebsiella pneumoniae*	>32	1	*SHV-1; TEM-1; CTX-M-55; KPC-2*	Favorable	Favorable	Alive
*Klebsiella pneumoniae*	>32	0.5	*SHV-1; TEM-1; CTX-M-55; KPC-2*	Favorable	Unfavorable	Alive
*Klebsiella pneumoniae*	>32	0.5	*SHV-1; TEM-1; CTX-M-55; KPC-2*	Favorable	Unfavorable	Alive
*Klebsiella pneumoniae*	2	0.12	*SHV-27; CTX-M-15*	Favorable	Favorable	Alive
*Klebsiella pneumoniae*	>32	1	*SHV-1; TEM-1; CTX-M-55; KPC-2*	Favorable	Favorable	Alive
*Klebsiella pneumoniae*	8	0.12	*KPC-2*	Favorable	Favorable	Alive
*Klebsiella pneumoniae*	4	0.12	*SHV-11; KPC-2*	Unfavorable	Indeterminate	Deceased
*Klebsiella aerogenes*	4	0.25	*TEM-1; CTX-M-15; KPC-2*	Favorable	Favorable	Alive
*Pseudomonas aeruginosa*	8	0.5	*PDC-24*	Favorable	Favorable	Alive
*Pseudomonas aeruginosa*	16	1	*PDC-56*	Favorable	Favorable	Alive
*Pseudomonas aeruginosa*	16	2	*PDC-3*	Favorable	Favorable	Alive
*Pseudomonas aeruginosa*	4	2	*PDC-3*	Favorable	Indeterminate	Deceased
*Pseudomonas aeruginosa*	16	2	*PDC-1*	Indeterminate	Indeterminate	Deceased
*Pseudomonas aeruginosa*	8	0.25	*PDC-98*	Favorable	Indeterminate	Deceased
*Acinetobacter calcoaceticus–baumannii* complex	4	2	N/A	Favorable	Favorable	Alive
*Haemophilus influenzae*	8	2	N/A	Favorable	Favorable	Alive
*Proteus mirabilis*	2	0.25	N/A	Favorable	Favorable	Alive

ACM, all-cause mortality; CTX, cefotaxime; EFU, early follow-up; EOT, end of treatment; IMI/REL, imipenem/cilastatin/relebactam; KPC, *Klebsiella pneumoniae* carbapenemase; LRT, lower respiratory tract; MIC, minimum inhibitory concentration; N/A, not available; OXA, oxacillinase; PDC, *Pseudomonas*-derived cephalosporinase; SHV, sulfhydryl reagent variable.

Molecular characterization of baseline LRT pathogens for the 18 participants with imipenem-nonsusceptible baseline pathogens found that half were *Klebsiella* spp. Among the nine *Klebsiella* spp., 67% carried *TEM-1*, 78% carried a sulfhydryl reagent variable (*SHV*) allele (five *SHV-1*, one each *SHV-11* and *SHV-27*), 78% carried *KPC-2*, 78% carried a cefotaxime (*CTX*)*-M* allele (four *CTX-M-55* and three *CTX-M-15*), and one *K. pneumoniae* isolate carried *OXA-48*. One *K. pneumoniae* isolate encoded both *SHV-11* and *KPC-2* and was susceptible to imipenem/REL at baseline (MIC = 0.12 µg/mL). This participant had died by the day 28 ACM assessment (Table 2). Six of the 18 LRT baseline imipenem-nonsusceptible pathogens (33.3%) were *P. aeruginosa*; four isolates encoded either *PDC-24*, *PDC-56*, *PDC-1*, or *PDC-98* (one each), while two isolates encoded *PDC-3*. There was one isolate each of *A. calcoaceticus–baumannii* complex, *H. influenzae*, and *Proteus mirabilis*, which were included based on MICs and the defined selection criteria, but did not meet requirements for molecular characterization described in S1 Fig.

Of 192 isolates from 56 participants treated with imipenem/REL, 59 gram-negative baseline isolates that were nonsusceptible to imipenem and related isolates from the same participants (NME [n = 26] and *P. aeruginosa* [n = 33]) were characterized molecularly (S1 Table and S1 Fig). Among the NME isolates, genes encoding ESBLs (n = 18 [69%]), KPC-2 (n = 8 [31%]), OXA-48-like (n = 6 [23%]), New Delhi metallo-β-lactamase-1 (*NDM-1*, n = 4 [15%]), and MIR-type AmpC (n = 1 [4%]) were found. Among the *P. aeruginosa* isolates, genes encoding MBL carbapenemases were observed in four (12%) isolates (two each carrying imipenemase 1 [*IMP-1*] or Verona integron‐encoded MBL 1 [*VIM-1* MBL]); 10 (30%) ESBL-encoding genes isolates, six carrying Vietnamese extended-spectrum β-lactamase (*VEB*) and four carrying pseudomonas extended resistance (*PER*), were also observed. *PDC-3* was the most frequent chromosomal AmpC allele encountered. In total, 152 isolates from baseline and subsequent visits were characterized molecularly for the presence of chromosomal and acquired β-lactamases using multiplex PCR and sequencing, of which 23 isolates qualified for relatedness testing (S2 Fig) and 35 isolates for porin analysis (S3 Fig).

Five participants with imipenem-susceptible and imipenem/REL-susceptible isolates at baseline had isolates collected at a later visit that yielded the same species, but no longer susceptible to either imipenem or imipenem/REL by CLSI susceptibility interpretive criteria (CLSI MIC ≤ 1 μg/mL for NME; MIC ≤ 2 μg/mL for *P. aeruginosa*) [16]. In three out of these five participants, pathogens with emergent imipenem/REL nonsusceptibility were identified (Table 3 and S4 Fig).

**Table 3:** MICs, molecular characterization, and corresponding clinical outcomes for baseline key LRT pathogens that were imipenem/REL-susceptible at baseline and became imipenem/REL-nonsusceptible during treatment with IMI/REL.

Participant	Time point	LRT pathogen	Imipenem MIC (µg/mL)	IMI/REL MIC (µg/mL)	Beta-lactamases	PFGE	Outer membrane entry porin	Microbiologic response at EOT	Clinical response at EFU	Day 28 ACM
1	BL	*Pseudomonas aeruginosa*	2	0.5	PDC-3	A	Full-length OprD	Unfavorable	Unfavorable	Alive
	EOT	*Pseudomonas aeruginosa*	32	8	PDC-3	B	No OprD PCR detected			
	EFU	*Pseudomonas aeruginosa*	32	4	PDC-3	B3	No OprD PCR detected			
2	BL	*Klebsiella pneumoniae*	≤0.5	0.12	SHV-11; TEM-1; CTX-M-15	A	Truncated OmpK35 (259 aa), full-length OmpK36 (369 aa)	Unfavorable	Unfavorable	Deceased
	OTX1	*Klebsiella pneumoniae*	32	32	SHV-11; TEM-1; CTX-M-15	B	Truncated OmpK35 (259 aa), full-length OmpK36 (369 aa)			
	OTX2	*Klebsiella pneumoniae*	>32	>32	SHV-11; TEM-1; CTX-M-15	B	Truncated OmpK35 (259 aa), full-length OmpK36 (369 aa)			
	OTX3	*Klebsiella pneumoniae*	2	2	SHV-11; TEM-1; CTX-M-15	A4	Truncated OmpK35 (259 aa), full-length OmpK36 (369 aa)			
	EOT	*Klebsiella pneumoniae*	2	2	SHV-11; TEM-1; CTX-M-15	A4	Truncated OmpK35 (259 aa), full-length OmpK36 (369 aa)			
3	BL	*Klebsiella pneumoniae*	1	1	SHV-11	A	OmpK35 (359 aa) and OmpK36 (365 aa	Favorable	Unfavorable	Alive
	Day 28	*Klebsiella pneumoniae*	4	4	SHV-1; TEM-1; CTX-M-15; OXA-48	B	OmpK35 (62 aa, C-terminally truncated) and OmpK36 (367 aa)			

aa, amino acid; ACM, all-cause mortality; BL, baseline; CTX, cefotaxime; EFU, early follow-up; EOT, end of treatment; IMI/REL, imipenem/cilastatin/relebactam; LRT, lower respiratory tract; MIC, minimum inhibitory concentration; OTX, on-treatment; OXA, oxacillinase; PCR, polymerase chain reaction; PDC, *Pseudomonas*-derived cephalosporinase; PFGE, pulsed-field gel electrophoresis; REL, relebactam; SHV, sulfhydryl reagent variable.

From Participant 1, three *P. aeruginosa* isolates identical by β-lactamase analysis, encoding PDC-3, were collected at baseline and at the EOT and EFU visits (Table 3). The baseline isolate harbored a full length 441-amino-acid-long OprD protein and was susceptible to both imipenem and imipenem/REL. The two isolates collected at the EOT visit were unrelated to the baseline isolate but were closely related to each other by PFGE analysis (S4 Fig). The *oprD* gene could not be amplified in either isolate, both of which were imipenem-nonsusceptible.

From Participant 2, a total of five *K. pneumoniae* isolates were collected (Table 3). Three isolates identical by β-lactamase and porin analysis and possibly related by PFGE analysis were collected at baseline and at the OTX3 and EOT visits (S4 Fig). The baseline isolate tested as susceptible to imipenem and imipenem/REL, whereas the two isolates collected at later visits tested with an intermediate MIC (interpreted as nonsusceptible) to both imipenem and impenem/REL (2 μg/mL). Two additional *K. pneumoniae* isolates that were unrelated to the baseline isolate but identical to each other by β-lactamase, porin, and PFGE analysis were collected at the OTX1 and OTX2 visits (S4 Fig). These two isolates were resistant to imipenem and imipenem/REL due to production of an NDM-1 metallo-β-lactamase, which relebactam does not inhibit.

From Participant 3, two *K. pneumoniae* isolates unrelated by β-lactamase, PFGE, and porin analysis were collected at baseline and day 28 (Table 3 and S4 Fig). The baseline isolate tested as susceptible to imipenem and imipenem/REL. The isolate collected at EOT tested as resistant to both imipenem and imipenem/REL (MIC = 4 μg/mL) due to the production of OXA-48.

## Discussion

The RESTORE-IMI 2 clinical study found that IMI/REL was non-inferior to PIP/TAZ for day 28 ACM and clinical response at EFU in the modified intent-to-treat population [15]. This subgroup analysis focused on outcomes among participants infected with imipenem-nonsusceptible pathogens for which relebactam restored *in vitro* susceptibility to imipenem and confirmed that outcomes were similar in this subpopulation of participants compared with participants infected with imipenem-susceptible pathogens. These results were expected, as relebactam was developed to restore the *in vitro* activity of imipenem among certain imipenem-resistant pathogens, such as KPC-producing Enterobacterales and imipenem-resistant *P. aeruginosa* [15,19]. Information on outcomes among resistant pathogens in Phase 3 indication-specific (e.g., HABP/VABP, cUTI, and cIAI) clinical studies is difficult to obtain owing to challenges with confirming resistant pathogens within an acceptable timeframe (i.e., within 24 or 48 hours of enrollment), and the low yield of resistant pathogens at participating study sites. Despite these difficulties, the subpopulation of patients infected with resistant pathogens represents the greatest area of unmet need and serves as the primary rationale for the development of novel antibacterial agents, such as IMI/REL [1–3]. The data described herein add to the available literature on the utility of IMI/REL for the treatment of carbapenem-resistant pathogens, as confirmed in the RESTORE-IMI 1 randomized controlled trial, in which IMI/REL was efficacious compared with colistin plus imipenem for the treatment of infections caused by imipenem-nonsusceptible pathogens [20].

The molecular mechanisms associated with the observed MICs of both imipenem and IMI/REL were included for descriptive purposes. No relationship between outcome and molecular profile was identified. Specifically, molecular characterization of imipenem-nonsusceptible isolates in this study found that genes encoding β-lactamases in NME isolates were mostly ESBLs ± AmpC (*CTX-M-15* and *MIR-type AmpC*), but *KPC-2*, *OXA-48-like*, and *NDM-1* were also present. In imipenem-nonsusceptible *P. aeruginosa* isolates, genes encoding MBL carbapenemases (*IMP-1* or *VIM-1*) were the most prevalent, followed by ESBL-encoding genes (*VEB* and *PER*). These carbapenemases represent some of the most frequent resistance mechanisms in carbapenem-resistant Enterobacterales and *P. aeruginosa* strains [21,22], and contribute to the increasing prevalence of difficult-to-treat infections worldwide [23–25].

IMI/REL has demonstrated efficacy against isolates recovered from difficult-to-treat infections [26,27], and this analysis showed potent *in vitro* activity against isolates carrying AmpC, KPC, and/or ESBL-encoding genes (100% susceptible by CLSI criteria). Reduced activity was observed against those isolates carrying genes encoding OXA-48-like β-lactamases (33% susceptible by CLSI criteria) or MBLs (0% susceptibility by CLSI criteria).

In our analysis, three participants with imipenem/REL-susceptible pathogens at baseline had pathogens isolated at later visits that were imipenem/REL-nonsusceptible. Differences in PFGE profiles of the isolates, as well as changes in enzyme carriage (in one case), indicate these are likely to represent unrelated secondary infections, rather than the emergence of resistance. Porin loss was evident for the *P. aeruginosa* from Participant 1, which may have contributed to the high imipenem MIC, while for Participant 3, the isolate collected 28 days post-randomization was differentiable from the baseline isolate by β-lactamase, PFGE, and porin analysis and was resistant to imipenem/REL due to production of the class D enzyme OXA-48. Of note, molecular characterization of isolates from Participant 2 revealed identical β-lactamase and porin profiles across isolates with marked differences in susceptibility to IMI/REL. One possible explanation for these differences could be the downregulation of *OmpK36* in the resistant isolates. OmpK36 has been shown to mediate the uptake of carbapenems across multiple strains of bacteria [28]. However, evaluation of porin expression levels was beyond the scope of our study. Of note, a second organism was isolated from Participant 1 (*Corynebacterium striatum*) and Participant 2 (*A. calcoaceticus–baumannii* complex).

Another study of five patients infected with *P. aeruginosa* strains that were initially imipenem-susceptible but became resistant found mutations in efflux operons in all five patients [29] in spite of genetic evidence in isogenic strains that neither imipenem nor relebactam are substrates for efflux pumps [19].

These results highlight the very low emergence of resistance in patients whose infecting pathogens are initially susceptible to imipenem and treated with IMI/REL. This subgroup analysis involved a small patient population, which could be considered a limitation of the study; however, this is also reflective of the relatively small number of individuals with HABP/VABP and confirmed carbapenem-nonsusceptible infections who meet eligibility requirements for clinical trials. There may be selection bias, which limits the applicability and generalizability of these results to other areas because of its small population size.

In summary, results from this subgroup analysis of RESTORE-IMI 2 support IMI/REL as a suitable treatment option for HABP/VABP caused by imipenem-nonsusceptible pathogens. It also provides evidence that the development of imipenem and imipenem/REL resistance as a result of IMI/REL treatment is rare. These results support the clinical utility of IMI/REL for the treatment of HABP/VABP caused by drug-resistant gram-negative pathogens.

## Supporting information

S1 Table β-lactamases identified in gram-negative baseline respiratory pathogens collected from participants in the mMITT population. (PDF)

S1 Fig Criteria for molecular characterization of β-lactamase genes. (PDF)

S2 Fig Algorithm to determine relatedness by PFGE when an imipenem/REL-NS or imipenem-NS isolate emerged during treatment. (PDF)

S3 Fig Algorithm for porin profiling of isolates. (PDF)

S4 Fig PFGE detection of the porin profile of isolates from the three participants with isolates that were imipenem/REL-susceptible at baseline but from whom imipenem/REL-NS pathogens were isolated during IMI/REL treatment. (PDF)
