# Supplementary material for: Imipenem/cilastatin/relebactam treatment of hospital-acquired/ventilator-associated bacterial pneumonia caused by imipenem-nonsusceptible pathogens: Subgroup analysis and molecular characterization of isolates from the RESTORE-IMI 2 clinical study
Source: PLoS One. 2026 Sep 15;21(9):e0357288. doi: 10.1371/journal.pone.0357288 (PMC13577331; doi:10.1371/journal.pone.0357288)
Supplement: Supplementary file 1 [file pone.0357288.s001.pdf]

S1 Table. β-lactamases identified in gram-negative baseline respiratory pathogens collected from participants in the mMITT population.

Organism/β-lactamase content	Number of isolates
Enterobacter cloacae	
MIR-TYPE	1
Escherichia coli	
CTX-M-15	1
Klebsiella aerogenes	
No acquired β-lactamases detected	1
TEM-1; CTX-M-15; KPC-2	1
Klebsiella pneumoniae	
SHV-11	1[a]
SHV-11; TEM-1; CTX-M-15	1[a]
SHV-27; CTX-M-15	1
KPC-2	1
SHV-11; KPC-2	1
SHV-1; TEM-1; CTX-M-55; KPC-2	5
SHV-11; OXA-48	1
SHV-11; CTX-M-15; OXA-48	1
SHV-1; TEM-1; CTX-M-15; OXA-48	2
SHV-11; TEM-1; CTX-M-15; OXA-48	2
SHV-1; CTX-M-55; NDM-1	1
SHV-28; TEM-1; CTX-M-15; NDM-1	2[b]
SHV-11; CTX-M-15; OXA-48; NDM-1	1
Serratia marcescens	
No acquired β-lactamases detected	2

Pseudomonas aeruginosa	
PDC-1	1
PDC-3	6[a,c]
PDC-5	2[b]
PDC-8	2
PDC-11	2[c]
PDC-12	1
PDC-14	1[a]
PDC-19A	1
PDC-24	1
PDC-56	1
PDC-98	1
PER-1; PDC-35	4
VEB-1A; PDC-11	3
VEB-9; PDC-11	2
VEB-16; PDC-35	1
PDC-12; IMP-1	2
PDC-3; VIM-2	2
Total number of subjects[b,c]	56

[a]Five respiratory pathogens isolated from five participants at baseline visits (one *Pseudomonas aeruginosa* carrying PDC-14; two *Pseudomonas aeruginosa* carrying PDC-3, one *Klebsiella pneumoniae* carrying SHV-11, and one *Klebsiella pneumoniae* carrying SHV-11, TEM-1, and CTX-M-15) tested as imipenem-susceptible by CLSI 2019 guidelines (MIC ≤1 µg/mL, Enterobacteriaceae; MIC ≤2 µg/mL, *Pseudomonas aeruginosa*). These organisms were screened for the presence of β-lactamase genes due to the collection of imipenem-nonsusceptible isolates of the same species from the same participant during the course of treatment.
[b]Two pathogens (one *Klebsiella pneumoniae* carrying SHV-28, TEM-1, CTX-M-15, and NDM-1 and one *Pseudomonas aeruginosa* carrying PDC-5) were isolated from one participant at the baseline visit.
[c]Three *Pseudomonas aeruginosa* pathogens carrying PDC-3 (n = 1) and PDC-11 (n = 2) were isolated from the same participant at the baseline visit.
CLSI, Clinical and Laboratory Standards Institute; CTX, cefotaxime; KPC, *Klebsiella pneumoniae* carbapenemase; MIC, minimum inhibitory concentration; mMITT, microbiological modified intention-to-treat population; NDM, New Delhi metallo-β-lactamase; OXA, oxacillinase; SHV, sulfhydryl reagent variable.
