# Supplementary material for: Imipenem/cilastatin/relebactam treatment of hospital-acquired/ventilator-associated bacterial pneumonia caused by imipenem-nonsusceptible pathogens: Subgroup analysis and molecular characterization of isolates from the RESTORE-IMI 2 clinical study
Source: PLoS One. 2026 Sep 15;21(9):e0357288. doi: 10.1371/journal.pone.0357288 (PMC13577331; doi:10.1371/journal.pone.0357288)
Supplement: Supplementary file 2 [file pone.0357288.s002.pdf]

Proprietary

S1 Figure. Criteria for molecular characterization of β-lactamase genes.

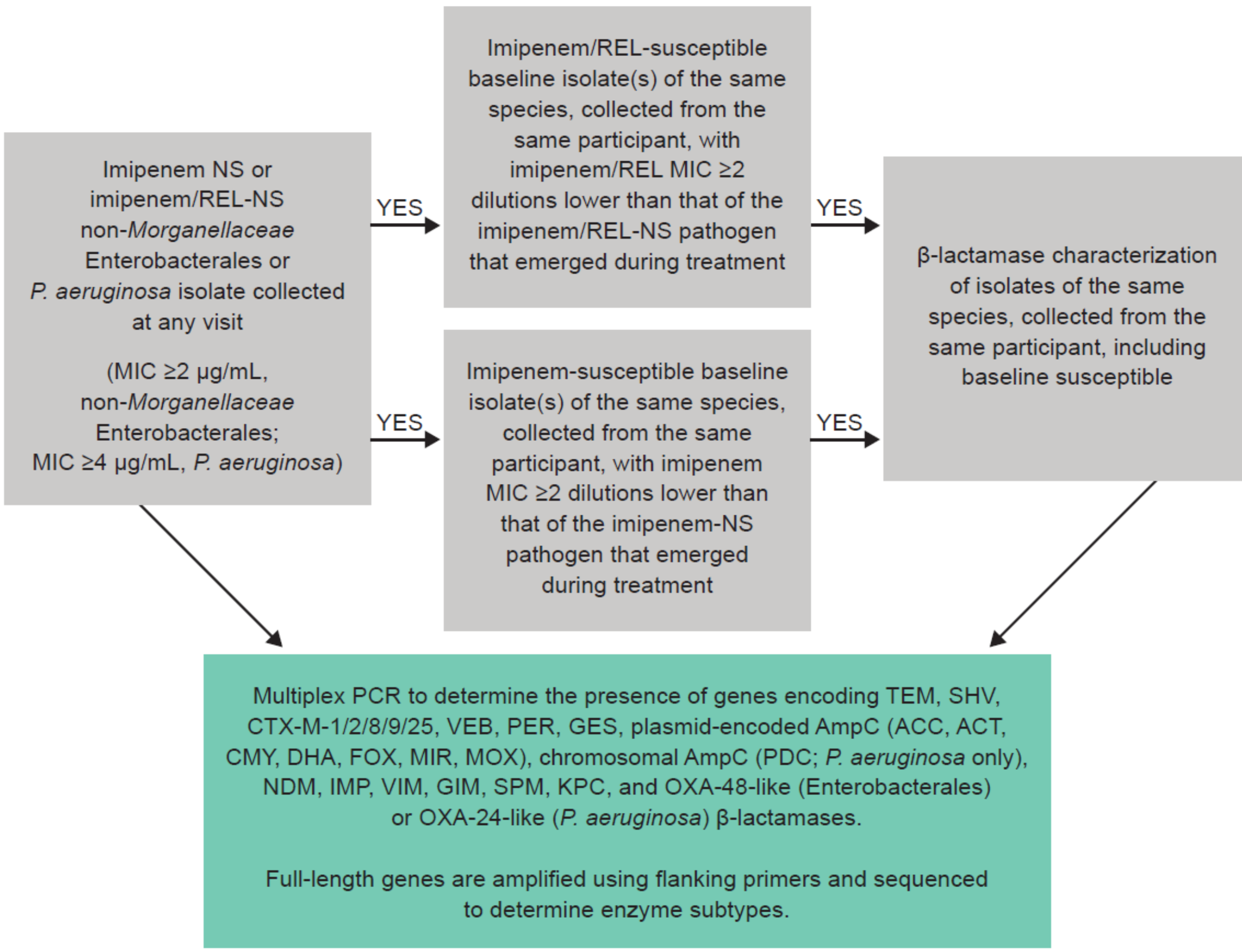

AmpC, ampicillin class C β-lactamase; CTX, cefotaxime; GES, Guiana extended-spectrum; GIM, German imipenemase; IMP, imipenemase; KPC, *Klebsiella pneumoniae* carbapenemase; MIC, minimum inhibitory concentration; NDM, New Delhi metallo-β-lactamase-1; NS, nonsusceptible; OXA, oxacillinase; *P. aeruginosa*, *Pseudomonas aeruginosa*; PCR, polymerase chain reaction; PDC, *Pseudomonas*-derived cephalosporinase; PER, pseudomonas extended resistance; REL, relebactam; SPM, São Paulo metallo-β-lactamase; SHV, sulfhydryl reagent variable; VEB, Vietnamese extended-spectrum β-lactamase; VIM, Verona integron-encoded metallo-β-lactamases.
