# Supplementary material for: Imipenem/cilastatin/relebactam treatment of hospital-acquired/ventilator-associated bacterial pneumonia caused by imipenem-nonsusceptible pathogens: Subgroup analysis and molecular characterization of isolates from the RESTORE-IMI 2 clinical study
Source: PLoS One. 2026 Sep 15;21(9):e0357288. doi: 10.1371/journal.pone.0357288 (PMC13577331; doi:10.1371/journal.pone.0357288)
Supplement: Supplementary file 3 [file pone.0357288.s003.pdf]

S2 Figure. Algorithm to determine relatedness by PFGE when an imipenem/REL-NS or imipenem-NS isolate emerged during treatment.

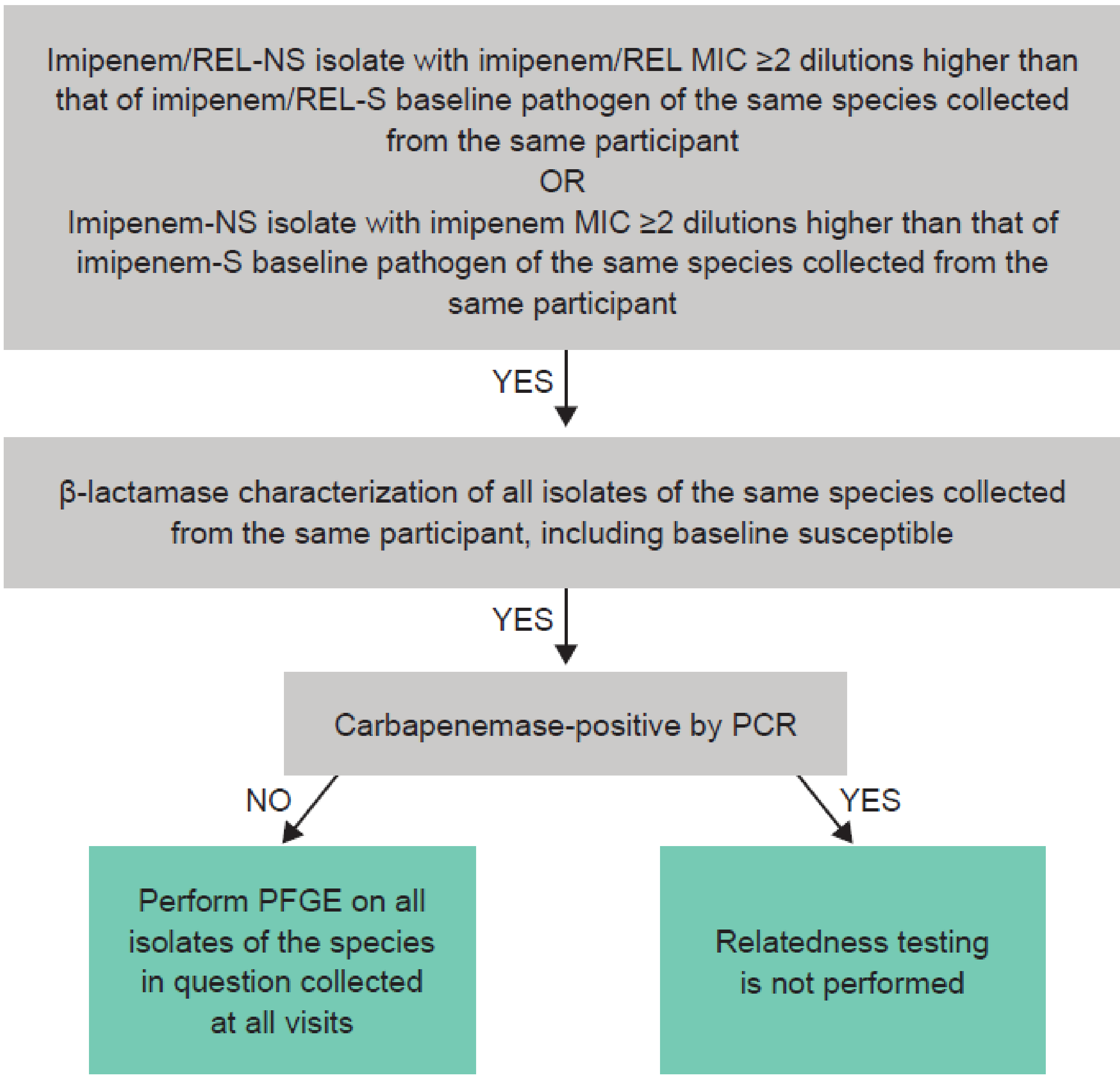

Tenover FC, et al. J Clin Microbiol. 1995;33:2233-2239.

MIC, minimum inhibitory concentration; NS, nonsusceptible; PCR, polymerase chain reaction; PFGE, pulsed-field gel electrophoresis; REL, relebactam; S, susceptible.
