# Supplementary material for: Imipenem/cilastatin/relebactam treatment of hospital-acquired/ventilator-associated bacterial pneumonia caused by imipenem-nonsusceptible pathogens: Subgroup analysis and molecular characterization of isolates from the RESTORE-IMI 2 clinical study
Source: PLoS One. 2026 Sep 15;21(9):e0357288. doi: 10.1371/journal.pone.0357288 (PMC13577331; doi:10.1371/journal.pone.0357288)
Supplement: Supplementary file 4 [file pone.0357288.s004.pdf]

S3 Figure. Algorithm for porin profiling of isolates.

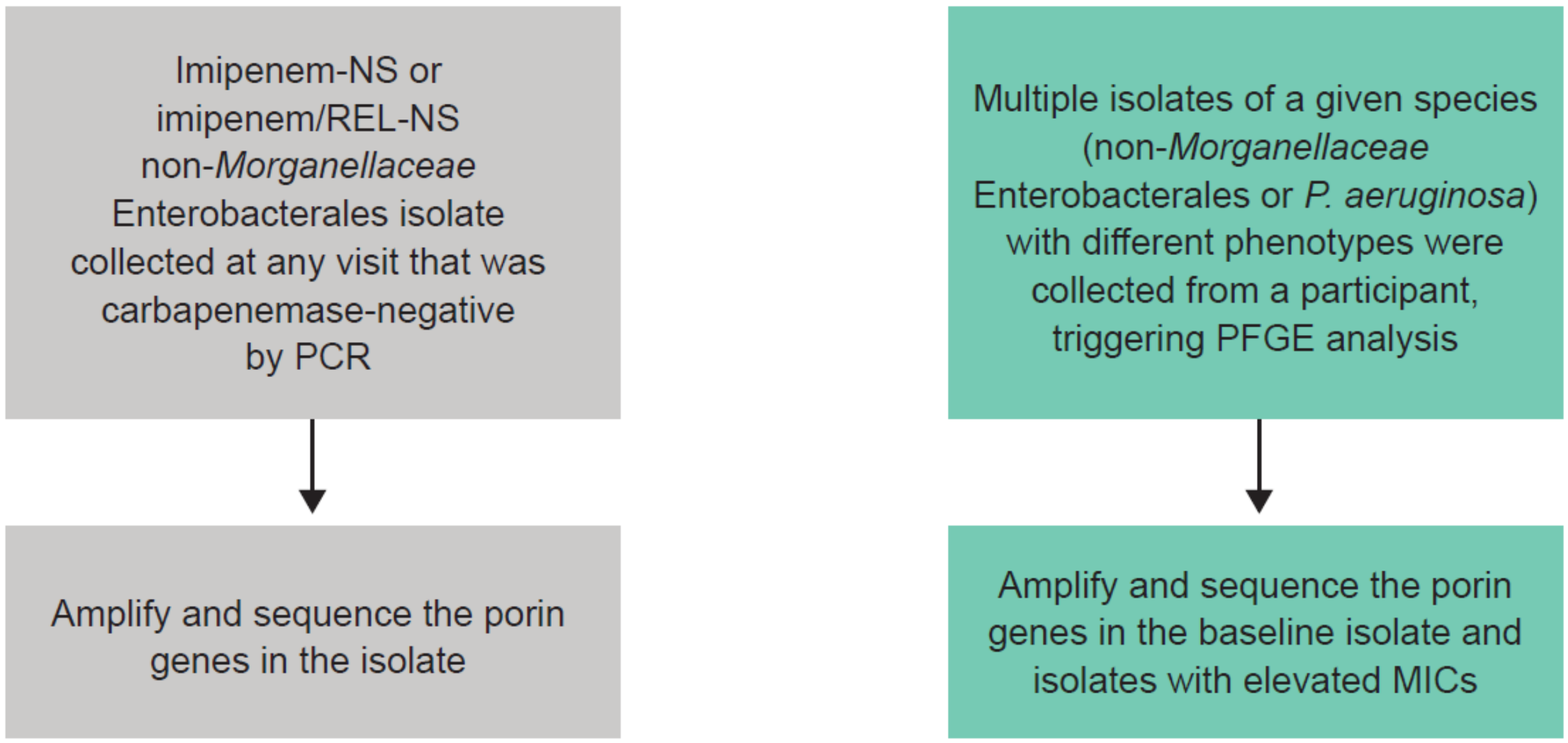

Wise MG, et al. J Med Microbiol. 2018;67:289-295.
MIC, minimum inhibitory concentration; NS, nonsusceptible; *P. aeruginosa*, *Pseudomonas aeruginosa*; PCR, polymerase chain reaction; PFGE, pulsed-field gel electrophoresis; REL, relebactam.
