# Supplementary material for: Imipenem/cilastatin/relebactam treatment of hospital-acquired/ventilator-associated bacterial pneumonia caused by imipenem-nonsusceptible pathogens: Subgroup analysis and molecular characterization of isolates from the RESTORE-IMI 2 clinical study
Source: PLoS One. 2026 Sep 15;21(9):e0357288. doi: 10.1371/journal.pone.0357288 (PMC13577331; doi:10.1371/journal.pone.0357288)
Supplement: Supplementary file 5 [file pone.0357288.s005.pdf]

S4 Figure original raw image:
P. aeruginosa (Participant 1)

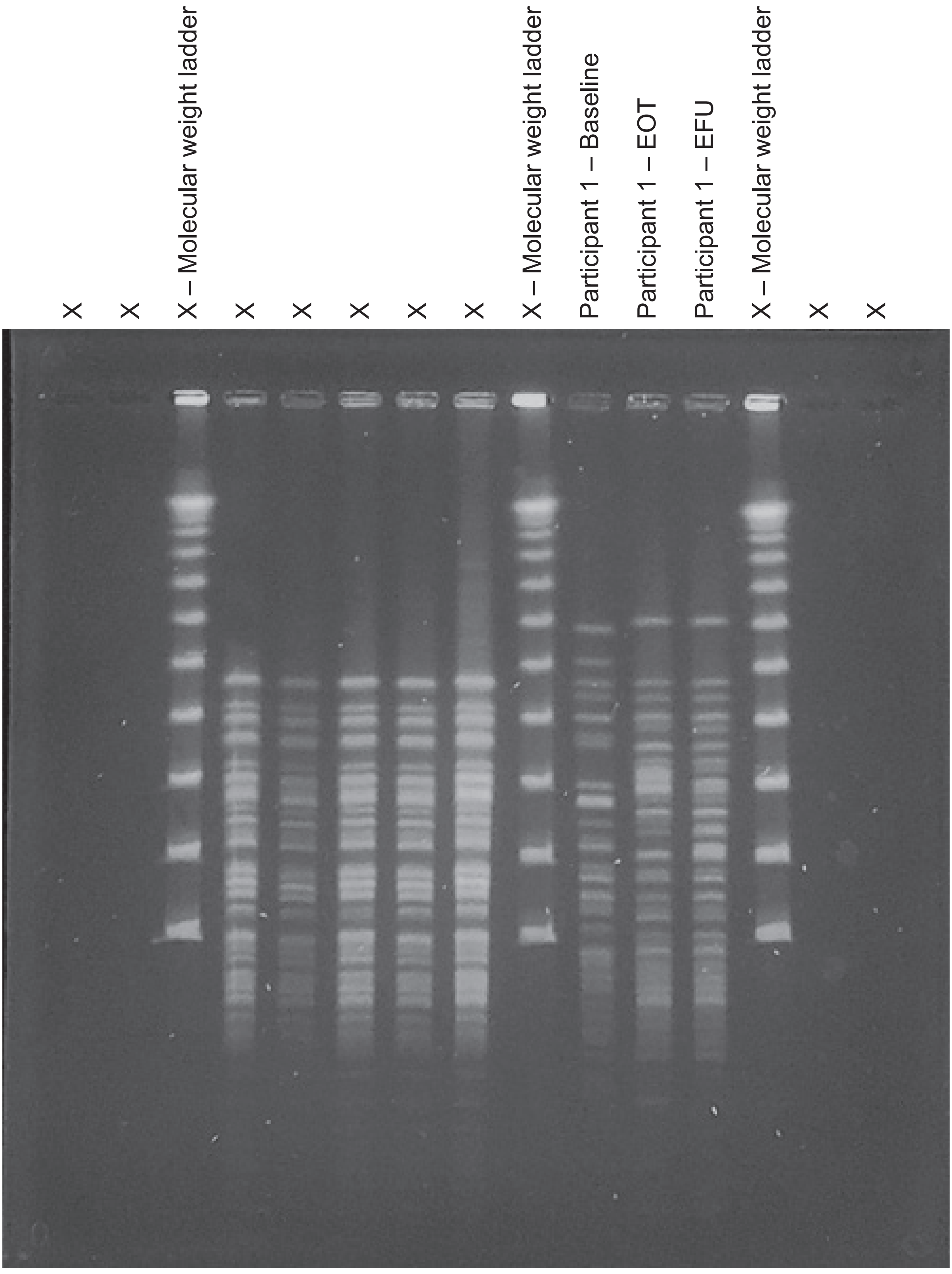

Image acquired with Apple iPhone.
EFU, early follow-up; EOT, end of treatment.

S4 Figure original raw image:
K. pneumoniae (Participants 2 and 3)

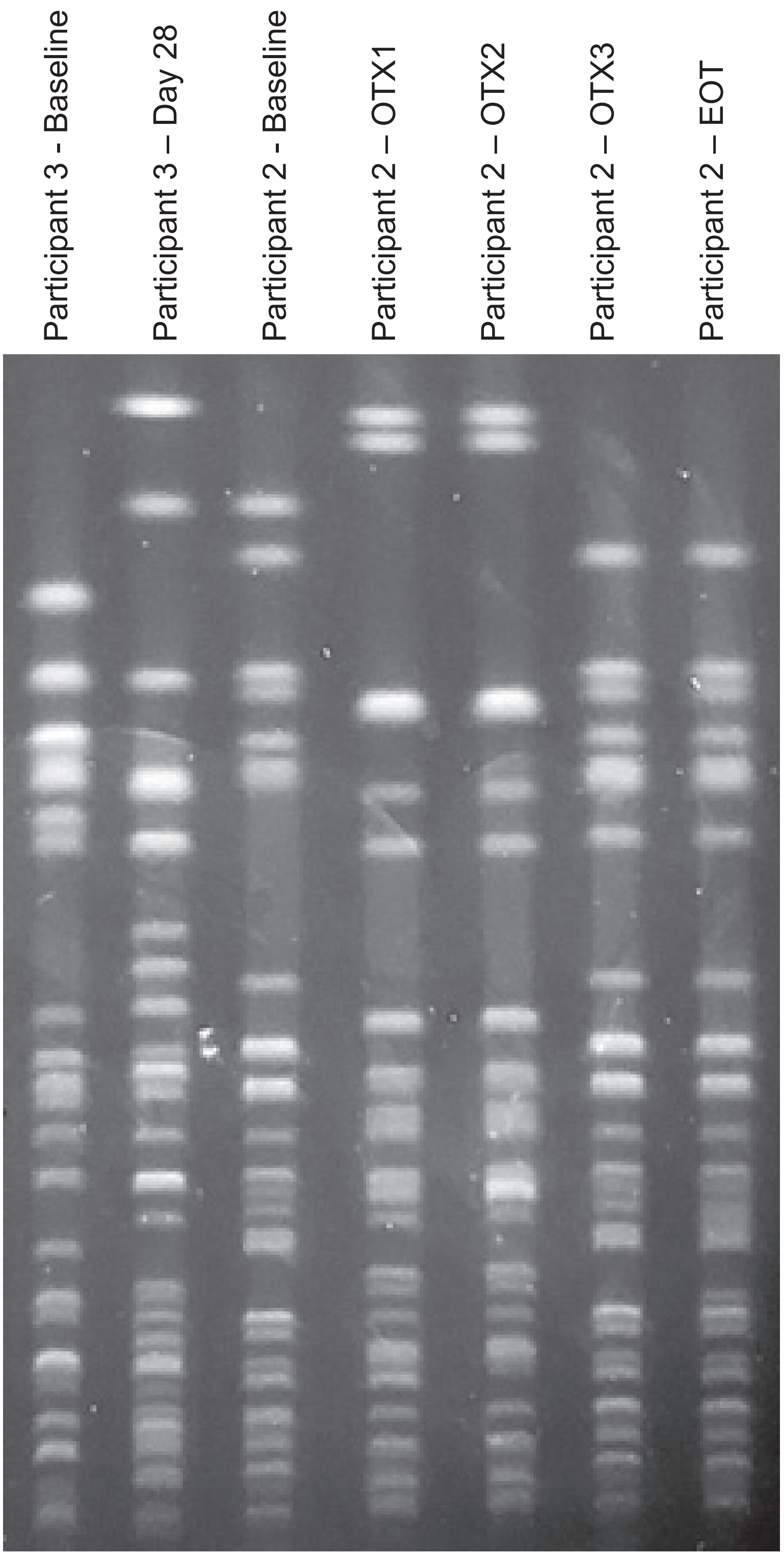

Image acquired with Apple iPhone.
EOT, end of treatment; OTX, on-treatment visit.
